# Assessing the incidence of complications and malignancies in the long-term management of benign biliary strictures with a percutaneous transhepatic drain

**DOI:** 10.1097/MD.0000000000029048

**Published:** 2022-03-11

**Authors:** Munehiro Yoshitomi, Ryuichi Kawahara, Shinichi Taniwaki, Ryuta Midorikawa, Satoki Kojima, Daisuke Muroya, Shoichiro Arai, Takahisa Shirahama, Hiroki Kanno, Shogo Fukutomi, Yuichi Goto, Yoriko Nomura, Masanori Akashi, Toshihiro Sato, Hisamune Sakai, Toru Hisaka, Yoshito Akagi

**Affiliations:** a Department of Surgery, Kurume University School of Medicine, Kurume, Fukuoka, Japan; b Department of Intensive Care, St Mary's Hospital, Kurume, Fukuoka, Japan.

**Keywords:** benign biliary strictures, long-term management, percutaneous transhepatic drain

## Abstract

Percutaneous drainage catheters (PDCs) are required for the management of benign biliary strictures refractory to first-line endoscopic treatment. While biliary patency after PDC placement exceeds 75%, long-term catheterization is occasionally necessary. In this article, we assess the outcomes of patients at our institution who required long-term PDC placement.

A single-institution retrospective analysis was performed on patients who required a PDC for 10 years or longer for the management of a benign biliary stricture. The primary outcome was uncomplicated drain management without infection or complication. Drain replacement was performed every 4 to 12 weeks as an outpatient procedure.

Nine patients (three males and six females; age range of 48–96 years) required a long-term PDC; eight patients required the long-term PDC for an anastomotic stricture and one for iatrogenic bile duct stenosis. A long-term PDC was required for residual stenosis or patient refusal. Drain placement ranged from 157 to 408 months. In seven patients, intrahepatic stones developed, while in one patient each, intrahepatic cholangiocarcinoma or hepatocellular carcinoma occurred.

Long-term PDC has a high rate of complications; therefore, to avoid the need for using long-term placement, careful observation or early surgical interventions are required.

## 1 Introduction

Percutaneous drainage catheter (PDC) placement for 6 to 12 months is an effective strategy for achieving permanent patency in the treatment of a benign biliary stricture in patients who are not surgical candidates or have failed endoscopic management.^[1]^ Twenty months after catheter removal, primary patency was reported in 81.25% of liver transplant recipients and 89.5% of all other patients.^[2]^ While all-comers statistics are encouraging,^[3]^ Weber et al^[4]^ in their management of 44 patients with benign anastomotic strictures after bilioenterostomy, who were therefore not endoscopy candidates, reported a success rate of PDC of 61.4% at a mean of 53.7 months of follow-up.

Long-term PDC may be the only option for patients who refuse surgical management or whose pathology or host characteristics make them poor surgical candidates. It is fraught with potential complications, including skin changes at the insertion site, poor quality of life, long-term radiation exposure during catheter exchanges,^[5]^ and chronic inflammatory changes in the biliary duct that may predispose to malignancy.^[6]^ Understanding long-term PDC risks is critical for assisting patients with informed decision-making and anticipating complications. However, few reported studies have evaluated these patients as a cohort. Thus, we aimed to evaluate our institutional experience with long-term PDC management of benign biliary stricture.

## 2 Methods

This is an institutional review board-approved retrospective analysis of patients who underwent PDC with chronic drain placement of >10 years at a single large academic medical center from April 1973 to March 2019.

The primary outcome was successful drain maintenance, which is defined as PDC without significant complication requiring an unplanned procedure against biliary tree malignancy development. Additional factors of interest included patient age and sex, PDC replacement interval, tube size, and length of drain placement.

This study was approved by the Institutional review board, Kurume University Hospital (approval no. 2021-012) and performed in accordance with the Declaration of Helsinki. The written informed consent requirement was waived off because of the retrospective design.

The descriptive statistics were calculated. All values were presented as mean ± standard deviation or ratio (percentage), unless otherwise stated. No comparative statistical analysis was performed.

## 3 Results

During the study period, a total of 2407 PDCs were placed. Of these, 34 were for a postoperative benign biliary stricture, 19 required a PDC for ≥1, and 9 had a drain for ≥10 years.

Subjects’ included three males and six females (age range: 48–96 years) with the following comorbidities: dementia (1); liver disease (severe: 1 and mild: 1); uncomplicated diabetes mellitus (2); Charlson's risk index (high: 1 and medium: 3).

The indication for PDC placement was an anastomotic stricture following cholangiojejunostomy in eight cases and iatrogenic bile duct stenosis in one case (Table 1). The reasons for the inability of drain removal included residual stenosis (8 patients) and patient refusal (1 patient).

**Table 1:** Nine cases PDC with chronic drain placement of >10 years.

Age	Condition	Surgery	Placement period (mo)	Replacement interval (wk)	Tube	Charlson risk index
48	Congenital biliary dilatation	Cholangiojejunostomy	408	6	18F internal fistula	
50	Congenital biliary dilatation	Cholangiojejunostomy	224	8	10F internal fistula	
63	Congenital biliary dilatation	Cholangiojejunostomy	351	3	16F internal fistula	Diabetes mellitus uncomplicated: 1
71	Congenital biliary dilatation	Cholangiojejunostomy	182	4	10F internal fistula	Liver disease severe: 3
71	Congenital biliary dilatation	Cholangiojejunostomy	227	4	12F internal fistula	
96	Cholelithiasis, bile duct injuries	Cholangiojejunostomy	178	8	16F internal fistula	Dementia: 1 diabetes mellitus uncomplicated: 1
67	Bile duct duodenal fistula	Cholangiojejunostomy, hepatectomy	157	8	14F internal fistula	Liver disease mild: 1
87	Cholelithiasis, bile duct injuries	Sphincteroplasty	275	2	18F internal fistula	
84	Neurilemoma	Cholangiojejunostomy	192	9	14F internal fistula	
						(High: 1/medium: 3)

Drain replacement was performed at a 4- to 12-week interval as an outpatient procedure (mean interval between exchanges: 5.8 ± 0.72 weeks). However, the other ten patients required a PDC for ≥1 and <10 years (mean interval between exchanges: 10.1 ± 2.31 weeks). Seven patients developed symptomatic intrahepatic stones for which percutaneous transhepatic cholangioscopic lithotripsy (PTCSL) was required one to three times. One patient developed an intrahepatic cholangiocarcinoma, while another developed hepatocellular carcinoma. Four representative cases are presented as follows.

### 3.1 Case 1

A 58-year-old male sustained a bile duct injury during cholecystectomy. The patient was referred to our institution following a sphincteroplasty and PDC placement. PDC exchange was performed every 8 weeks. A biliary stricture and intrahepatic stones developed at the age of 61, 64, and 72, requiring PTCSL on each occasion. At the age of 79, the patient had frequent episodes of cholangitis with increasing biliary sludge volumes observed during each PDC exchange (Fig. S1, Supplemental Digital Content), requiring frequent drain exchanges. Moreover, repeat biliary duct imaging showed sclerotic changes and stenosis in places other than the initially injured places (Fig. S2, Supplemental Digital Content). The patient had repetitive cholangitis episodes due to tube obstruction, and tube replacement became more frequent. Internal fistula management became impossible, and we endeavored to control the cholangitis with an external fistula, performing drain replacements once every 3 to 4 weeks. At the age of 87, the patient died from a malignant urological condition.

### 3.2 Case 2

**Figure 1:**
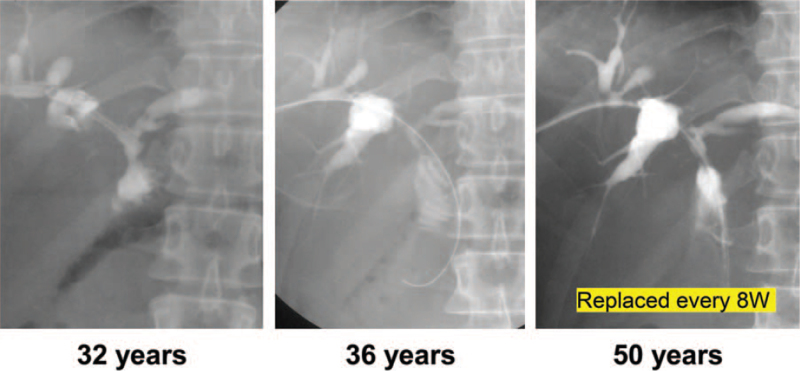
Bile duct imaging revealed stenosis of the intrahepatic bile duct.

A 19-year-old female underwent extrahepatic bile duct resection and cholangiojejunostomy for congenital biliary dilatation. At the age of 32, an anastomotic stricture and intrahepatic stones developed, requiring PDC and PTCSL. At the age of 36, she required PTCSL for the second time for intrahepatic stones. However, no improvement in the patient's anastomotic stricture was observed and gradual intrahepatic bile duct stenosis developed (Fig. 1). PDC exchanges were performed every 12 weeks; however, currently, her drain clogs due to the biliary sludge more frequently and replacements are required every 6 to 8 weeks. Although surgery was considered, the patient refused the operative management and wished to continue with regular drain replacements. This continues at the age of 50, 31 years after her initial PDC placement.

### 3.3 Case 3

**Figure 2:**
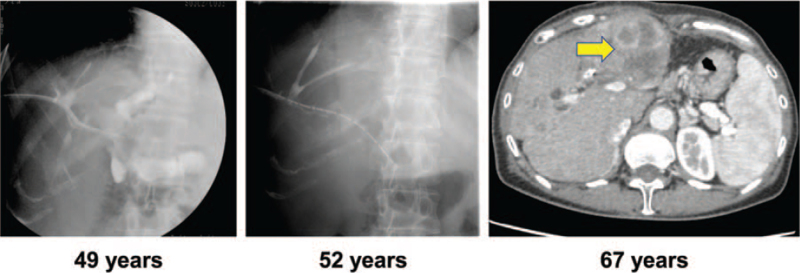
Few bile ducts were visible on cholangiography. In a contrast-enhanced CT scan, an intrahepatic cholangiocarcinoma was visible in the residual left lobe of the liver.

A 30-year-old female underwent hepatectomy and cholangiojejunostomy due to intrahepatic stones. At the age of 48, PDC and PTCSL were first performed. While removals were attempted, PDC was performed twice more and remained in place. At the age of 67, the patient had repetitive episodes of refractory cholangitis (Fig. 2). Since distinguishing the cholangitis from an abscess was difficult, a contrast-enhanced computed tomography (CT) scan and liver biopsy were performed, and intrahepatic cholangiocarcinoma was diagnosed. Unfortunately, her disease was unresectable. After 3 months of attempted multidisciplinary treatment with chemotherapy without improvement, the patient transitioned to palliative care and died. In this particular case, bile cytodiagnosis came back negative. This occurred despite the close observation for 10 years due to concern for cancer.

### 3.4 Case 4

**Figure 3:**
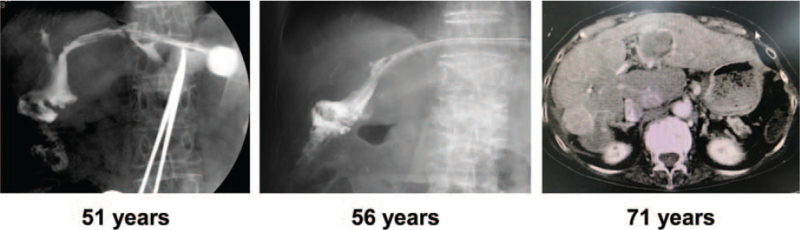
No bile ducts were initially visible on cholangiography. In a contrast-enhanced CT scan, multiple deposits of hepatocellular carcinoma in both lobes of the liver were visible.

A 43-year-old female underwent extrahepatic bile duct resection and cholangiojejunostomy for congenital biliary dilatation at another hospital. At the age of 50, she developed an anastomotic stricture and intrahepatic stones, requiring PDC and PTCSL. At that point, no causal viral infection or liver cirrhosis was evident and there was a secondary biliary cirrhosis accompanied by cholestasis, making surgical treatment difficult. Her stenosis did not improve, requiring chronic PDC management. At the age of 71, she developed hepatocellular carcinoma (Fig. 3), but cancer treatment was unable to be performed because of her poor hepatic functional reserve. The patient transitioned to palliative care and passed away shortly after that.

## 4 Discussion

Although PDC placement is largely effective for a period of 6 to 12 months, selected patients may require long-term or indefinite therapy due to surgical contraindication, distorted anatomy, or their own preference. In a retrospective analysis of our experience with nine patients who required PDC for >10 years, we observed a high rate of intrahepatic stone formation (77.8%) and malignancy (22.2%).

Over the course of patient therapy, more frequent PDC exchanges (exchange frequency: 5.8 weeks) were required, which was significantly shorter than the 12 ± 4.5-week interval previously reported by Weber et al.^[4]^ More frequent PDC changes appeared to be related to an increased biliary sludge volume noted on the PDC tube during exchanges. The increased sludge formation is a sequela of secondary sclerosing cholangitis, which is commonly reported in long-term biliary obstructions.^[7]^ Damage to the biliary tree from a prior surgery or the benign biliary stricture itself could compromise the biliary tree's natural defenses against potentially toxic bile salts. The resulting increase in inflammatory cytokines released from damaged endothelium can lead to further progressive tissue damage and hepatic injury.^[8]^ As per the results of the previous studies, the mean follow-up period was 4 to 5 years without these sequelae; therefore, it appears that a significant duration of chronic biliary tree inflammation is required before the onset of this condition. However, the sequelae of secondary sclerosing cholangitis include hepatic damage^[8]^ and an increased risk of malignancy.^[9]^ As many of the patients in our series had no other viable option in the management of their benign biliary stricture other than PDC, we hypothesize that reducing the degree of bile acid buildup may result in improved long-term outcomes or at least delay the progression of chronic inflammatory changes.

While the surgical management of benign biliary strictures has improved, perioperative and late complication rates of up to 25% have been reported.^[2,10]^ These rates discouraged us from performing surgery in one patient in whom an external fistula used at a late stage resulted in a significant decrease in her quality of life. Furthermore, endoscopic management of benign biliary strictures is recommended whenever possible while most of our patients were not surgical candidates because of anatomical reasons, the outcomes of this work reveal that chronic PDC should be avoided wherever possible. The risks of surgery versus the risks of chronic PDC should be discussed with patients. Furthermore, it should be emphasized that long-term damage related to chronic PDC and secondary sclerosing cholangitis may not permit surgery in the future.

Carcinogenesis was observed in two cases (22.22%) in our series. In addition to hepatolithiasis,^[11–13]^ chronic bile duct damage/inflammation,^[10]^ and cholangiojejunostomy^[11,14]^ are known risk factors for intrahepatic cholangiocarcinoma. The possibility that PDCs themselves also contributed to this proinflammatory milieu cannot be discounted. However, it is likely that any impact of the PDC minimally affected the vicious cycles of inflammation that existed before and during PDC therapy. In our patient group, more vigilant surveillance could have enabled earlier cancer detection; however, surveillance alone cannot prevent cancer, and no preventative options were available. We hypothesize that the key to managing the oncologic risks of patients with long-term PDCs is controlling the cholestasis and secondary sclerosing cholangitis resulting from significant biliary sludge buildup. While definitive management with hepatectomy and biliary tract reconstruction was not viable for patients in our series, we believe that pilot studies involving medical therapies, such as those for biliary dyspepsia, may be important in improving the longevity and quality of life of patients who require long-term care with PDC.

In addition to its nature as a retrospective study, this study has several notable limitations. It includes a small sample size to evaluate a rather rare clinical indication. Therefore, the patients in this cohort poorly represent the entire population of patients who require long-term PDC. Furthermore, the treatment period of these patients spanned decades, during which multiple advances have occurred in minimally invasive endoscopy for benign biliary strictures. Finally, without a treatment group, we have only historical comparisons to evaluate our complication and malignancy rates. While a prospective trial is unlikely, given the rarity of the modern indications for chronic PDC, a multicenter retrospective analysis with demographic and pathology-matched controls would significantly improve the interpretability and reliability of our findings.

In conclusion, we report a high rate of complications and malignancy in the chronic management of benign biliary sclerosis with PDC. Therefore, alternative management should be considered whenever possible. Future assessment of potential ways to improve the longevity and quality of life of patients who can only be managed with PDC is required.

## Acknowledgments

The authors thank Crimson Interactive Pvt. Ltd (Ulatus)—www.ulatus.jp for their assistance in manuscript translation and editing. The authors would like to thank all the doctors who specialize in PTBD at Kurume University School of Medicine.

## Author contributions

Acquisition of data: Shinichi Taniwaki, Ryuta Midorikawa, Satoki Kojima, Daisuke Muroya, Shoichiro Arai, Takahisa Shirahama.

All authors approved the final version of the manuscript and agreed to be accountable for all aspects of the work in ensuring that questions related to the accuracy or integrity of any part of the work are appropriately investigated and resolved.

Analysis and interpretation of data: Hiroki Kanno, Shogo Fukutomi, Yuichi Goto, Yoriko Nomura, Masanori Akashi, Toshihiro Sato, Hisamune Sakai.

Critical revision: Ryuichi Kawahara, Toru Hisaka, Yoshito Akagi.

Drafting the manuscript: Munehiro Yoshitomi.

Study conception and design: Munehiro Yoshitomi, Ryuichi Kawahara, Toru Hisaka, Yoshito Akagi.

**Conceptualization:** Munehiro Yoshitomi, Ryuichi Kawahara, Toru Hisaka, Yoshito Akagi.

**Data curation:** Shinichi Taniwaki, Ryuta Midorikawa, Satoki Kojima, Daisuke Muroya, Shoichiro Arai, Takahisa Shirahama.

**Formal analysis:** Hiroki Kanno, Shogo Fukutomi, Yuichi Goto, Yoriko Nomura, Masanori Akashi, Toshihiro Sato, Hisamune Sakai.

**Investigation:** Hiroki Kanno, Shogo Fukutomi, Yuichi Goto, Yoriko Nomura, Masanori Akashi, Toshihiro Sato, Hisamune Sakai.

**Writing – original draft:** Munehiro Yoshitomi.

**Writing – review & editing:** Ryuichi Kawahara, Toru Hisaka, Yoshito Akagi.

## Supplementary Material

Supplemental Digital Content

## Supplementary Material

Supplemental Digital Content
