# Supplementary material for: Assessing the incidence of complications and malignancies in the long-term management of benign biliary strictures with a percutaneous transhepatic drain
Source: Medicine (Baltimore). 2022 Mar 11;101(10):e29048. doi: 10.1097/MD.0000000000029048 (PMC8913096; doi:10.1097/MD.0000000000029048)

**Supplemental Information**

***Assessing the Incidence of Complications and Malignancies in the Long-Term Management of*** ***Benign Biliary Strictures with a*** ***Percutaneous Transhepatic Drain***

Munehiro Yoshitom, et al.

**Supplemental Figure**

Supplemental Fig. 1. Bile sludge on a withdrawn PDC.


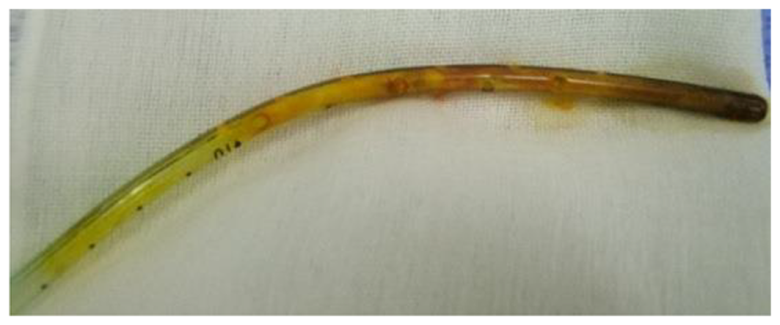

Supplement: Supplemental Digital Content [file medi-101-e29048-s001.doc]
